# *In situ* fabrication of a graphene-coated three-dimensional nickel oxide anode for high-capacity lithium-ion batteries†‡

**DOI:** 10.1039/c7ra10987c

**Published:** 2018-02-14

**Authors:** Chiwon Kang, Eunho Cha, Sang Hyub Lee, Wonbong Choi

**Affiliations:** Department of Materials Science and Engineering, University of North Texas Discovery Park 3940 North Elm St. Denton TX 76207 USA wonbong.choi@unt.edu; IBS Center for Integrated Nanostructure Physics, Institute for Basic Science, Sungkyunkwan University Suwon 16419 South Korea; Department of Energy Science, Department of Physics, Sungkyunkwan University Suwon 16419 South Korea; Department of Mechanical and Energy Engineering, University of North Texas Discovery Park 3940 North Elm St. Denton TX 76207 USA

## Abstract

The high theoretical specific capacity of nickel oxide (NiO) makes it attractive as a high-efficiency electrode material for electrochemical energy storage. However, its application is limited due to its inferior electrochemical performance and complicated electrode fabrication process. Here, we developed an *in situ* fabrication of a graphene-coated, three-dimensional (3D) NiO–Ni structure by simple chemical vapor deposition (CVD). We synthesized NiO layers on Ni foam through a thermal oxidation process; subsequently, we grew graphene layers directly on the surface of NiO after a hydrogen-assisted reduction process. The uniform graphene coating renders high electrical conductivity, structural flexibility and high elastic modulus at atomic thickness. The graphene-coated 3D NiO–Ni structure delivered a high areal density of ∼23 mg cm^−2^. It also exhibits a high areal capacity of 1.2 mA h cm^−2^ at 0.1 mA cm^−2^ for its Li-ion battery performance. The high capacity is attributed to the high surface area of the 3D structure and the unique properties of the graphene layers on the NiO anode. Since the entire process is carried out in one CVD system, the fabrication of such a graphene-coated 3D NiO–Ni anode is simple and scalable for practical applications.

## Abstract 2

The processing of graphene coated NiO–Ni anode using one CVD system delivered high Li-ion battery performance.

## Graphical abstract

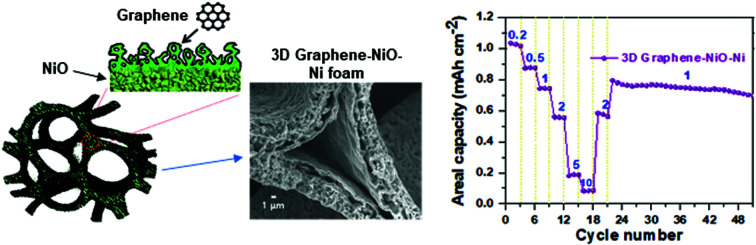

## 1. Introduction

The increasing demand for high-efficiency, large-scale electrochemical energy storages (*e.g.*, electric vehicles) has led to an expansion in new developmental efforts for high energy-density lithium-ion batteries (LIBs).^1^ However, commercial graphite anodes involving the conventional Li ion (Li^+^) intercalation reaction have a low theoretical specific capacity (372 mA h g^−1^), which prevents them from being applied in advanced energy storage.^2^ In this context, transition metal oxides have been considered promising electrode materials because of their high theoretical capacities, chemical stability and low cost.^3,4^ Furthermore, the conversion reaction of 2*y*Li^+^ + M_*x*_O_*y*_ ↔ *x*M + *y*Li_2_O (M denotes transition metals such as Ni, Cu, Fe, and Co) is a thermodynamically favorable reaction that facilitates large amount of electron transfers, which leads to two- to three-fold superior capacity (600–1100 mA h g^−1^).^5^ Among transition metal oxides, nickel oxide (NiO) could be a strong contender due to its higher theoretical specific capacity (718 mA h g^−1^), chemical stability, environmental benignity and low cost.^6^ Nevertheless, the inherently low electrical conductivity (*ρ* > 10^15^ Ωm at room temperature) and low specific surface area hinder NiO from achieving high LIB performance.^7^ Furthermore, the conversion reaction of NiO–Li_2_O poses another problem associated with the large volume change between NiO and Li_2_O during cycling.^4^ Therefore, our focus in this study is specifically on the following two: first is altering the structural properties (*e.g.*, morphology, size and porosity) of NiO to increase its Li^+^ ion diffusion rate, surface-to-volume ratio and structural stability for enhanced electrochemical performance;^8–10^ second is incorporating a conducting element into NiO to enhance its electrical and mechanical properties.^11–19^ In particular, the integration of graphene into NiO has received great attention owing to unique features of graphene such as superior electrical conductivity,^20,21^ structural flexibility and high elastic modulus at atomic thickness.^22–32^ Various methods to prepare NiO–graphene nanocomposite structures include, but are not limited to, hydrothermal synthesis,^23–27^ core–shell spray pyrolysis,^28^ nanoparticles-sheet assembly,^29,30^ ultrasonication^31^ and electrical wire pulse technique.^32^ Overall, the resultant NiO–graphene structures show specific capacity (700–1098 mA h g^−1^) superior to NiO (100–439 mA h g^−1^). However, such a high specific capacity has never been translated into increased areal capacity (capacity per footprint area), which is crucial for practical application in LIBs. The low areal capacity has been considered a critical drawback in most nanomaterial-based anodes. Nevertheless, no attention has been paid to enhancing areal capacity of NiO–graphene anode for large-scale, advanced LIB.

Our previous experiment demonstrated how 3-dimensional (3D) structures could enhance the areal capacity of electrodes in LIBs.^33,34^ By following this concept, we fabricated a novel structure of graphene-coated 3D NiO–Ni anode through a simple two-step thermal chemical vapor deposition (CVD) method, in which graphene layers were grown directly on a NiO–Ni structure. In this structure, porous 3D Ni substrate offered high surface area to accommodate large loading of NiO; in addition, the porous structure facilitated lithium ion diffusion within NiO.^8,9,15,16^ The *in situ* graphene growth on NiO was achieved by a simple CVD process right after reduction of the NiO process in the same CVD chamber;^23–32^ the process was effective, yet facile, to produce a highly stable graphene network throughout the 3D NiO structure. The graphene-coated 3D NiO–Ni anode delivered improved areal density (∼23 mg cm^−2^) and higher areal capacity (1.2 mA h cm^−2^ at 0.1 mA cm^−2^) than previously reported NiO-based anodes;^8–10,12–15,18,19^ such values are critical for practical applications. The excellent properties and novel design of the graphene coated 3D NiO–Ni anode would expand the development of large-scale LIBs.

## 2. Experimental

### 2.1. Synthesis of graphene-coated 3D NiO–Ni electrode

**Fig. 1:**
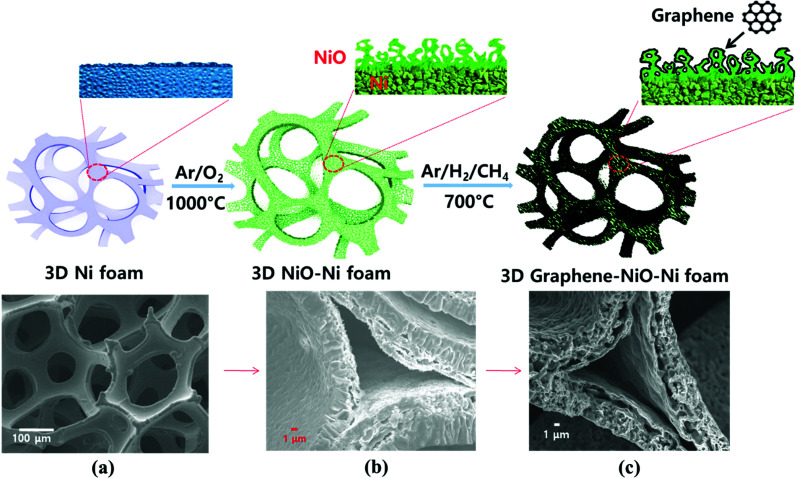
Schematic representation of the steps to synthesize 3D graphene–NiO–Ni with the corresponding cross-sectional models and their corresponding field emission scanning electron microscope (FESEM) images: (a) structure of porous Ni foam; (b) NiO grown on the porous Ni foam by a thermal CVD oxidation process; (c) graphene grown on the NiO–Ni foam by a two-step process of H_2_-assisted reduction of NiO and subsequent graphene growth.

Porous Ni foam, with nominal cell size of 450 μm and porosity of 85% (Alantum), was used as a pristine substrate for NiO growth. The Ni foam was cut and then inserted into a quartz tube of a thermal chemical vapor deposition (CVD) system (Atomate). For NiO growth, the substrate was rapidly heated, in an Ar (500 sccm) and O_2_ (125 sccm) mixed gas environment (4 : 1 volume ratio), to a temperature of 1000 °C for 2 hours. At the final stage, the as-grown NiO on Ni foam was naturally cooled to room temperature within the tube under inert Ar gas atmosphere. After the growth process, the gray Ni foam was transformed into a greenish stoichiometric NiO structure. Subsequently, the CVD tube was rapidly heated to 700 °C within the Ar gas environment. Once temperature was reached, the mixture of CH_4_, H_2_, and Ar gases was introduced at flow rates of 50, 100 and 500 sccm (1 : 2 : 10 volume ratio), respectively. During the graphene growth process of 1 minute, the thermally decomposed carbon from the precursor CH_4_ gas was absorbed onto the reduced Ni from NiO (the NiO reduction process simultaneously occurred by H_2_ gas). Consequently, a graphene-coated NiO–Ni nanocomposite structure was synthesized. Fig. 1 schematically illustrates the fabrication procedures for the graphene coated 3D NiO–Ni foam.

### 2.2. Structural characterization

The morphologies of the NiO–Ni foam and graphene-coated NiO–Ni foam structures were identified with a field emission scanning electron microscope (FESEM) (JEOL, JSM-7000F). Elemental analysis for both structures was carried out using an energy dispersive spectroscope (EDS) (FEI Helios 650). For cross-sectional SEM-EDS analysis, the samples were frozen in liquid nitrogen (77 K) and then cut into two pieces. The structural property of the samples was also characterized with an X-ray diffractometer (XRD) (Rigaku, Rint-2000) using Cu K-alpha radiation in the range of 10–90° (2*θ*) with step size 0.01° and with a Raman spectroscope (Jobin-Yvon, Labram HR) using Ar^+^ laser with *λ* = 514 nm and 0.5 mW power.

### 2.3. Electrochemical characterization

A CR2032 coin cell (Wellcos Ltd.) was assembled with as-grown 3D graphene–NiO–Ni as a working electrode and lithium foil as both counter and reference electrodes. No current collector or additive was incorporated into the assembled anode; this is advantageous for enhanced energy and power density of an LIB cell (for example, deadweight of conventional current collector constitutes nearly 10% of the total weight of an LIB cell^35,36^). 1.0 M LiPF_6_ in ethylene carbonate–dimethylene carbonate–diethylene carbonate (EC–DMC–DEC) (1 : 1 : 1 in volume) and a typical polypropylene (PP) based membrane (Separator-2400, Wellcos Ltd.) served as an electrolyte and a separator, respectively. The complete cell assembly was conducted in an argon-filled glovebox that maintained oxygen and humidity levels less than 0.5 ppm. The charge–discharge cycling behaviors of the cell were characterized with a multi-channel battery tester (MACCOR-series 4000) in galvanostatic mode (constant current). In this study, charge and discharge processes were related to the oxidation and reduction (conversion) reactions as NiO + 2Li^+^ + 2e^−^ ↔ Ni + Li_2_O, respectively. The cells were cycled in the voltage range of 0.01–3.0 V at different current densities. Cyclic voltammetry (CV) measurements for the 3D graphene–NiO–Ni and 3D NiO–Ni anode samples were conducted using a multi-channel potentiostat (Bio Logic, VMP3) in the voltage range of 0.01 to 3.0 V (*vs.* Li^+^/Li) at a scan rate of 0.1 mV s^−1^.

## 3. Results and discussion

### 3.1. Structural characterization of NiO–Ni foam

**Fig. 2:**
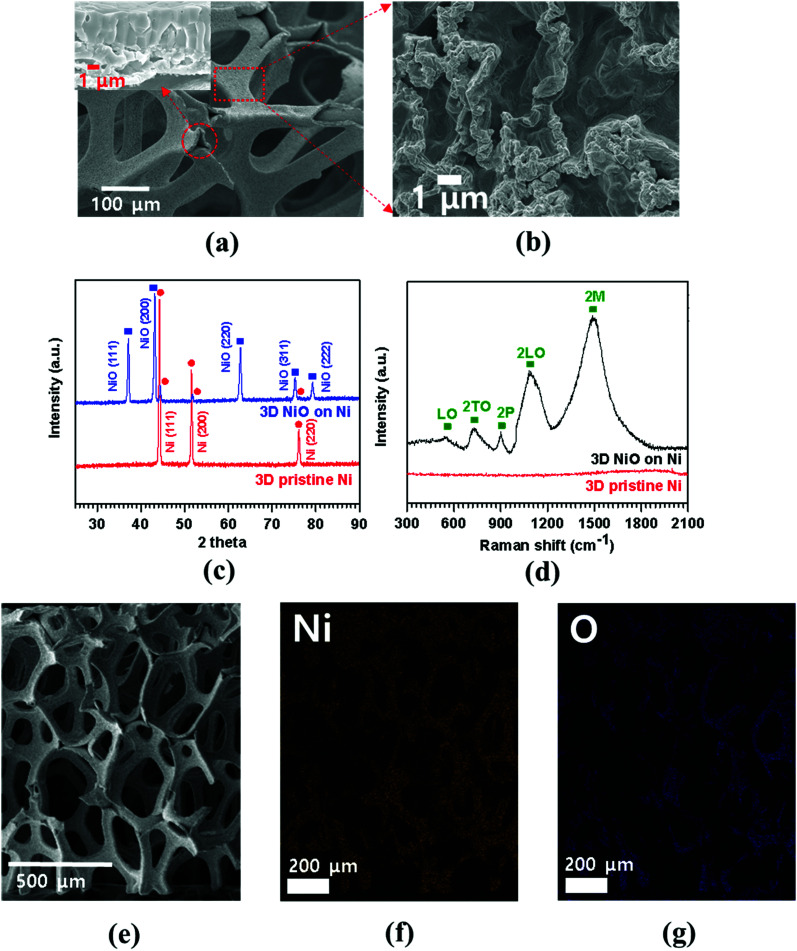
(a, b) Low and high magnification FESEM images demonstrating the surface morphologies of a thermally grown 3D porous NiO on Ni, respectively (the inset of (a) illustrates a cross-sectional image of 3D NiO on Ni with high magnification); (c) XRD patterns and (d) Raman spectra of the 3D NiO–Ni hybrid and pristine 3D Ni structures; (e) SEM image of 3D NiO–Ni for EDS mapping; (f, g) EDS mapping results from nickel and oxygen elements comprising 3D NiO–Ni structure, respectively.

FESEM image of porous Ni foam showed the average pore size was 150 μm and the width was approximately 40 μm (Fig. 1(a)) with smooth polycrystalline surface. After thermal oxidation, as-synthesized porous NiO was uniformly grown throughout the Ni surface while preserving the micro-channeled structure (Fig. 2(a)).^17^Fig. 2(a and b) demonstrate low and high magnification FESEM images showing disordered sub-micron NiO nanoparticles on the surface of Ni foam.^3,4,8,9^ Moreover, the cross-sectional images display columnar structured NiO layers grown on porous Ni foam (Fig. 1(b) and its enlarged FESEM image is included in an inset of Fig. 2(a)). The growth mechanism of NiO on Ni foam is dictated by the thermal diffusion and reaction of Ni^2+^ and O^2−^ ions in Ni foam according to the Kirkendall effect.^37–39^ Thermally induced volume expansion facilitates outward diffusing of Ni^+^ ions through grain and grain boundaries of crystalline NiO, thus forming columnar NiO structures. The X-ray diffraction (XRD) pattern confirms the evolution of NiO phase grown on Ni by thermal CVD oxidation (Fig. 2(c)). The strong intense XRD peaks appeared at 37.1°, 43.2°, 62.7°, 75.3° and 79.3°, which corresponded to the crystallographic plane indices of (1 1 1), (2 0 0), (2 2 0), (3 1 1) and (2 2 2) for a cubic NiO phase, respectively (JCPDF card 47-1049). The average size of the NiO crystallites was about 27.2 nm by Scherrer equation; this value is comparable to the previously reported NiO nanoparticles in their constituent porous NiO.^40^ In addition, the presence of the remaining Ni phase in the 3D NiO–Ni after thermal oxidation was manifested by intense XRD peaks observed at 44.2°, 51.6° and 76.1° corresponding to (1 1 1), (2 0 0) and (2 2 0) for Ni, respectively (JCPDF card 4-850). Note that the presence of the Ni phase has the advantage of enhancing electrical conductivity of NiO and catalytic activity that facilitates decomposition of Li_2_O and formation of the solid electrolyte interphase (SEI) layer during the charging process.^4^ No other peaks relevant to the impurities were identified in the XRD patterns. The Raman spectra further corroborate the results from the XRD analysis (Fig. 2(d)). A typical one-phonon peak at ∼570 cm^−1^ (LO mode), three two-phonon peaks at ∼730 cm^−1^ (2TO mode), ∼906 cm^−1^ (TO + LO mode) and ∼1090 cm^−1^ (2LO mode), and one strong two-magnon peak at ∼1490 cm^−1^ (2M mode) were observed; the peaks were consistent with previous results from NiO.^41^ In contrast, no Raman peak for Ni indicates the lack of active vibrational Raman mode in Ni.^9^ In Fig. 2(e–g), the EDS elemental mapping images represent the distribution of Ni and oxygen (O) elements on the 3D NiO–Ni. Noticeably, an average areal density of NiO only in the 3D structure is empirically measured as ∼23 mg cm^−2^. As evident in Fig. S1,‡ the weight ratio of 3D NiO (∼51%) as an electroactive material to 3D NiO–Ni is consistent with the compositional ratio of ∼50% NiO. These are the highest values reported to date by the thermal oxidation process.^8–10,12–19^ The average areal density is obtained by the formula *m*_NiO_ = Δ*m* × 149.38/32 for the reaction of 2Ni + O_2_ = 2NiO, where *m*_NiO_ is the weight of NiO, Δ*m* (the weight of O) is the real weight difference between NiO and Ni after NiO growth, and 149.38 and 32 g mol^−1^ are the molecular weights of 2NiO and O_2_, respectively.^11^ Therefore, we could confirm that our exerted oxidation condition is more intense than other reported ones that processed the Ni–NiO structures.^8–10,12–19,28^

### 3.2. Structural characterization of graphene coated NiO–Ni foam

**Fig. 3:**
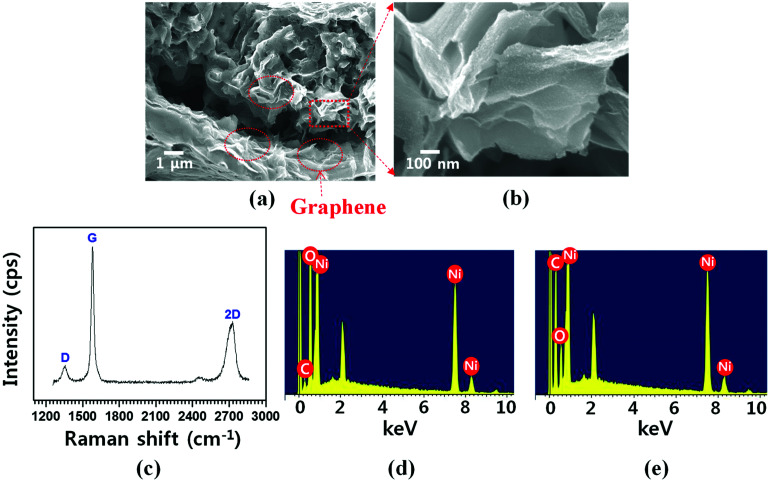
Cross-sectional FESEM images of characteristic 3D graphene grown on porous NiO structure with (a) low and (b) high magnifications, respectively; (c) Raman spectra that identify graphene on NiO structure in terms of D-, G- and 2D-band characteristic peaks. EDS spectra reveal constituting elements in (d) 3D NiO–Ni and (e) 3D graphene–NiO–Ni.

Thermal CVD is more facile for direct growth of high-quality graphene on metal substrates (*e.g.*, Cu and Ni); additionally, it allows excellent physico-chemical properties of graphene.^22,23,31^ In principle, graphene is not directly grown on NiO due to insolubility of carbon into NiO.^42^ Thus, the H_2_ reduction process is required to transform NiO to Ni for graphene growth. The amount of O vacancies in the NiO structure increases with elevated temperature during the reduction process and catalyzes cleavage of the hydrogen bond (H–H); thus, the reaction produces H_2_O gas and leaves behind a Ni structure.^43^ Graphene growth proceeded right after the NiO reduction process by using the same CVD system.^44^ The process steps are summarized as follows: (1) 3D NiO–Ni structure was annealed in Ar environment at up to 700 °C. (2) CH_4_/H_2_ (1 : 2 volume ratio) gas mixture was introduced into the reactor. (3) The structure was cooled to room temperature in Ar environment. In step (2), hydrocarbon (*e.g.*, methane in this study) is thermally decomposed; subsequently, the resultant carbon atoms dissolved into Ni after NiO is transformed into Ni by H_2_ reduction process. During step (3), carbon atoms are segregated and then precipitated on the Ni surface; thus, graphene layers are grown.^45^Fig. 3(a and b) show the cross-sectional FESEM images demonstrating the graphene-coated 3D NiO–Ni structure. In the images, the presence of graphene is evidenced by the characteristic rippled and wrinkled structures.^46^ The presence of the as-grown graphene is also confirmed by the typical D peak at 1355 cm^−1^, G peak at 1581 cm^−1^ and 2D peak at 2706 cm^−1^ in the Raman spectra (Fig. 3(c)).^47^*I*_D_/*I*_G_ peak ratio (∼0.2) and *I*_2D_/*I*_G_ (∼0.5) are indicative of high-quality and multi-layered graphene. Note that peaks corresponding to NiO structure are not observed due to the screening effect caused by graphene on the graphene/NiO surface. This is evidenced by the Raman spectra with wavenumber ranging from 300 to 1000 cm^−1^, where no NiO peaks are observed (Fig. S2‡). Furthermore, fewer graphene layers were grown on NiO by decreasing the concentration of carbon precursor gas (C_2_H_4_) during graphene synthesis; thus, LO-mode peak for NiO at ∼490 cm^−1^ is observed in the Raman spectra (Fig. S3‡). The presence of graphene is further confirmed by the intense carbon peak in the EDS spectra (Fig. 3(e)). The weak carbon (C) peak for NiO–Ni (Fig. 3(d)) is presumed to be artifacts (*i.e.*, carbon conductive tape). The areal density of graphene (∼0.17 mg cm^−2^) was measured by the weight difference of the sample before and after CVD growth.

### 3.3. Lithium-ion battery performance

**Fig. 4:**
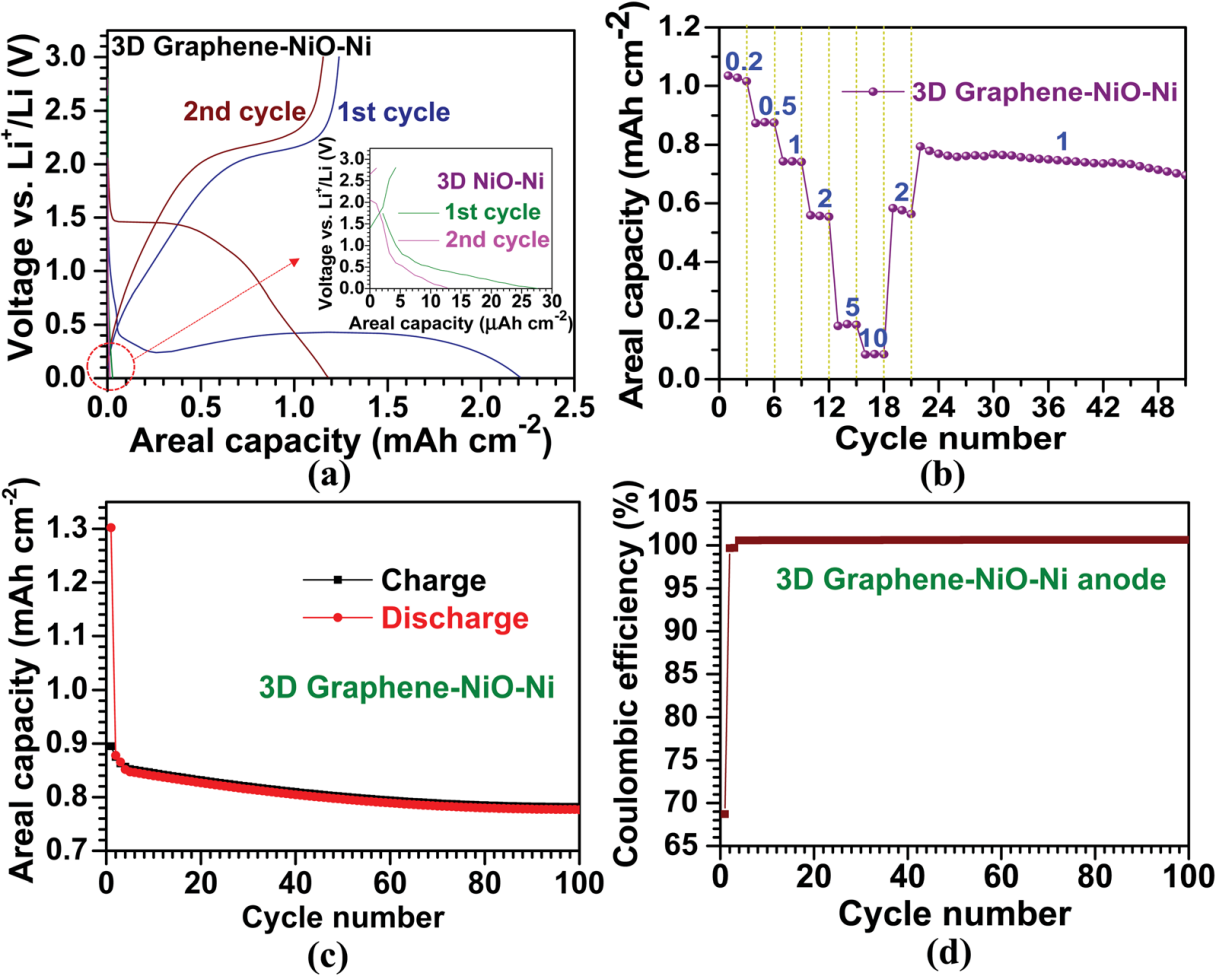
Electrochemical performance of the 3D graphene–NiO–Ni and 3D NiO–Ni anodic materials; (a) characteristic voltage profiles of the 3D graphene–NiO–Ni and 3D NiO–Ni for the first two cycles; (b) C-rate capability of the 3D graphene–NiO–Ni anode at the six different current densities; the denoted numbers represent the applied current densities with a unit of mA cm^−2^; (c) cycling performance and (d) coulombic efficiency of the 3D graphene–NiO–Ni anode at 1 mA cm^−2^ for 100 cycles.

The electrochemical properties of the 3D graphene–NiO–Ni working electrode were tested using a CR2032 coin-type half-cell. Fig. 4(a) illustrates voltage *vs.* areal capacity profiles for the first two cycles at a current density of 0.1 mA cm^−2^. For the first discharge of the 3D graphene–NiO–Ni anode, an extended plateau region (∼1.5 mA h cm^−2^) was observed between 0.25 V and 0.5 V. The plateau region is ascribed to the formation of solid electrolyte interphase (SEI) on the surface of the 3D graphene–NiO–Ni through electrolyte decomposition; it is also due to the reduction from Ni^2+^ to Ni^0^ by Li^+^ ion uptake into NiO based on NiO + 2Li^+^ ↔ Li_2_O + Ni, forming Li_2_O.^4,23^ It should be noted that the voltage fluctuation at the plateau region might come from the large amount of SEI formation at the graphene–electrolyte interface. Nanostructured materials are commonly susceptible to their unstable or irreversible capacity loss that occurs in the first cycle.^48^ The gradual increase of the first charge profile up to 2.0 V shows the plateau region of 2.0–2.1 V, in which oxidation reaction from Ni^0^ to Ni^2+^ occurred, forming NiO.^23,30^ Notice that coulombic efficiency (the ratio of charge (1.2 mA h cm^−2^) to discharge (2.2 mA h cm^−2^) capacities) for the first cycle at 0.1 mA cm^−2^ is measured as ∼55% (derived from Fig. 4(a)), similar to other reports of graphene–NiO hybrid anodes.^23–25,27,29,31,32^ However, coulombic efficiency increases up to ∼98% acquired by the ratio of charge (1.16 mA h cm^−2^) to discharge (1.18 mA h cm^−2^) capacities from the second cycle. The gradual decrease in voltage profile for the discharge process was observed in 1–1.5 V; the profile is related to the reduction reaction from Ni^2+^ to Ni^0^.^4,23^ The second discharge curve exhibited the voltage plateau region at ∼1.5 V; the higher voltage over the first discharge is closely related to the large variation of NiO microstructure and texture involved in the irreversible formation of Li_2_O and the decomposition of SEI layer formed during the first cycle.^4^ For the second charge, the voltage profile was similar to that of the first charge except with a slightly higher plateau voltage range of 2.1–2.2 V due to increased anodic polarization in a cell.^30^ The overall voltage–capacity curves showed similar profiles to the previously reported NiO-based anodes.^4,15,17,23,30^ Our prepared 3D NiO–Ni anode before graphene growth demonstrates different voltage profiles with negligibly low areal capacities (<0.01 mA h cm^−2^ in the inset of Fig. 4(a)) which were seemingly due to cell resistance from a high NiO weight ratio (∼51% in our experiments). These results ensured that graphene grown on 3D NiO–Ni structure contributed to such improvement in LIB performance by providing efficient conducting pathways among NiO phase regions and structural buffers against structural strains induced by large volume variations of NiO during cycling. Therefore, a facile electronic transfer from bulk electrode to electroactive NiO nanomaterials was achieved^23,32,49–51^ while the structural integrity of 3D NiO was preserved.^23,32^ The cyclic voltammetry (CV) curves for the 3D graphene–NiO–Ni and 3D NiO–Ni anode samples are displayed in Fig. S4.‡ It is noted that 3D graphene–NiO–Ni demonstrates peaks corresponding to NiO (two anodic peaks at ∼1.7 and ∼2.2 V, respectively, and a cathodic peak at ∼1.3 V) and graphene (a cathodic peak at ∼0.01 V). These results are in line with the voltage *vs.* capacity profiles of Fig. 4(a).

Moreover, Fig. 4(b) demonstrates the cycling performance of the LIB cell for the 3D graphene–NiO–Ni as a function of current density. The areal capacities are 1, 0.9, 0.7, 0.6, 0.2 and 0.1 mA h cm^−2^ at 0.2, 0.5, 1, 2, 5 and 10 mA cm^−2^, respectively; the values are higher than those from other reported NiO anodes.^18,19^ The average areal capacity of the anode (0.6 mA h cm^−2^), after running through 10 mA cm^−2^, recovered to the former value of 2 mA cm^−2^ (higher than 99% capacity retention); this confirmed high structural integrity and rate capability of our proposed 3D graphene–NiO–Ni anode. The subsequent cycling performance at 1 mA cm^−2^ resulted in an average areal capacity of 0.75 mA h cm^−2^, which is ∼140% higher than the previous reports on nanoscale NiO anodes;^18,19^ thus, the resulting capacity retention (∼90%) indicated excellent cell stability. Furthermore, cycling stability of the anode at 1 mA cm^−2^ for 100 cycles is presented in Fig. 4(c); the overall coulombic efficiency of the anode is nearly 100% after the first cycle (69%) (Fig. 4(d)). Based on the promising LIB performance, we confirmed that graphene served as an important electrical conducting and structural buffering agent for the NiO, which addressed pitfalls of NiO such as its insulating nature and capacity loss induced by large volume variation during cycling. Additionally, the self-supporting 3D graphene–NiO–Ni structure required no binder, current collector or conducting agent (*e.g.*, carbon black) for anode fabrication. Such materials will act as inefficient deadweight constituents.^33^ Although 3D graphene–NiO–Ni anode demonstrated improved areal capacity, gravimetric specific capacity could not be acquired due to the difficulty in determining weight fraction among the three components of the anode (*i.e.* graphene, NiO and Ni) after the CVD processing. Nevertheless, as mentioned earlier, no one has yet applied the current CVD approach to enhance the areal capacity of NiO and LIB efficiency with graphene. Thus, the importance of our results from the CVD approach would be realized by its implementation into practical applications for large-scale LIBs and other energy storage systems.

## 4. Conclusion

We have fabricated a novel 3D graphene–NiO–Ni anode *via* a simple two-step thermal CVD method to increase its areal density up to ∼23 mg cm^−2^. Such value provides a higher amount of active materials for LIBs which is important for large-scale practical applications. The *in situ* graphene grown on the reduced 3D NiO–Ni exhibits wrinkled, high-quality, and multi-layered structures. Such growth is a simple and highly effective method for large-scale coating of graphene onto a nano-porous electrode. While graphene layer grown on NiO is effective as an electrically conducting and structurally buffering for the nano-porous NiO, the 3D graphene–NiO–Ni anode exhibits a high rate capability with an areal capacity of 1.2 mA h cm^−2^ at 0.1 mA cm^−2^. Our 3D graphene–NiO–Ni anode could be applied to ever-expanding development of large-scale, advanced LIBs.

## Conflicts of interest

There are no conflicts of interest to declare.

## Supplementary Material

RA-008-C7RA10987C-s001
