# Supplementary material for: In situ fabrication of a graphene-coated three-dimensional nickel oxide anode for high-capacity lithium-ion batteries
Source: RSC Adv. 2018 Feb 14;8(14):7414–21. doi: 10.1039/c7ra10987c (PMC9078377; doi:10.1039/c7ra10987c)
Supplement: RA-008-C7RA10987C-s001 [file RA-008-C7RA10987C-s001.pdf]

Electronic Supplementary Material (ESI) for RSC Advances.
This journal is © The Royal Society of Chemistry 2018

Supplementary Data

In-Situ Fabrication of Graphene Coated Three-Dimensional Nickel Oxide Anode for High Capacity Lithium-Ion Batteries

Chiwon Kang[1,†], Eunho Cha[1,†], Sang Hyub Lee[2,3], Wonbong Choi[1,4*]

[1*]Department of Materials Science and Engineering, [4]Department of Mechanical and Energy Engineering, University of North Texas, Discovery Park 3940 North Elm St., Denton, TX 76207, USA

[2]IBS Center for Integrated Nanostructure Physics, Institute for Basic Science, [3]Department of Energy Science, Department of Physics, Sungkyunkwan University, Suwon 16419, South Korea

[†]These two authors equally contributed.

[*]E-mail: wonbong.choi@unt.edu

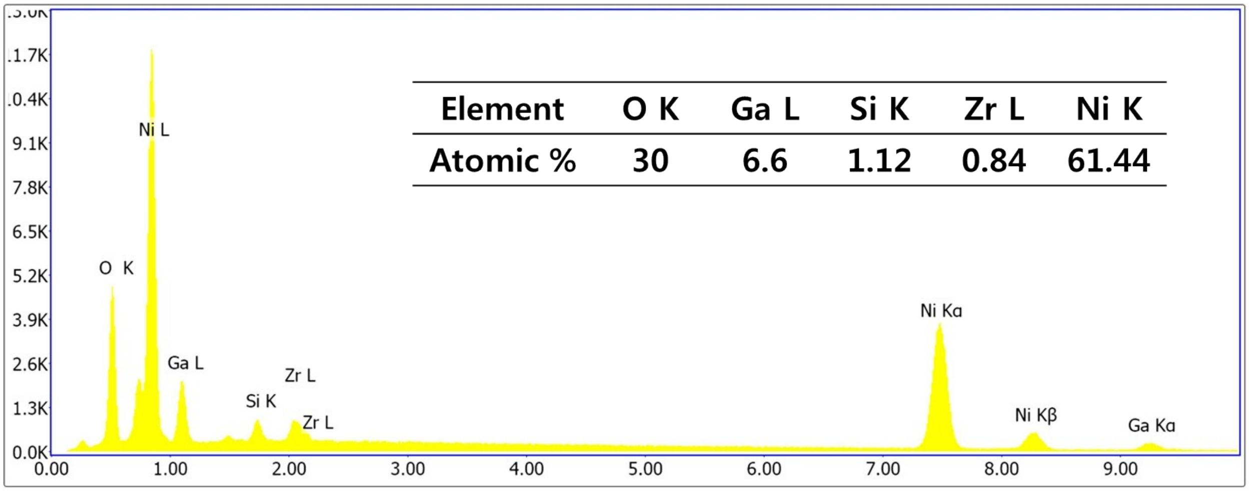

Element	O K	Ga L	Si K	Zr L	Ni K
Atomic %	30	6.6	1.12	0.84	61.44

Figure S1. EDS spectra for the constituent elements of the 3D NiO-Ni. Note that the Ga peak is artifact from the FIB process.

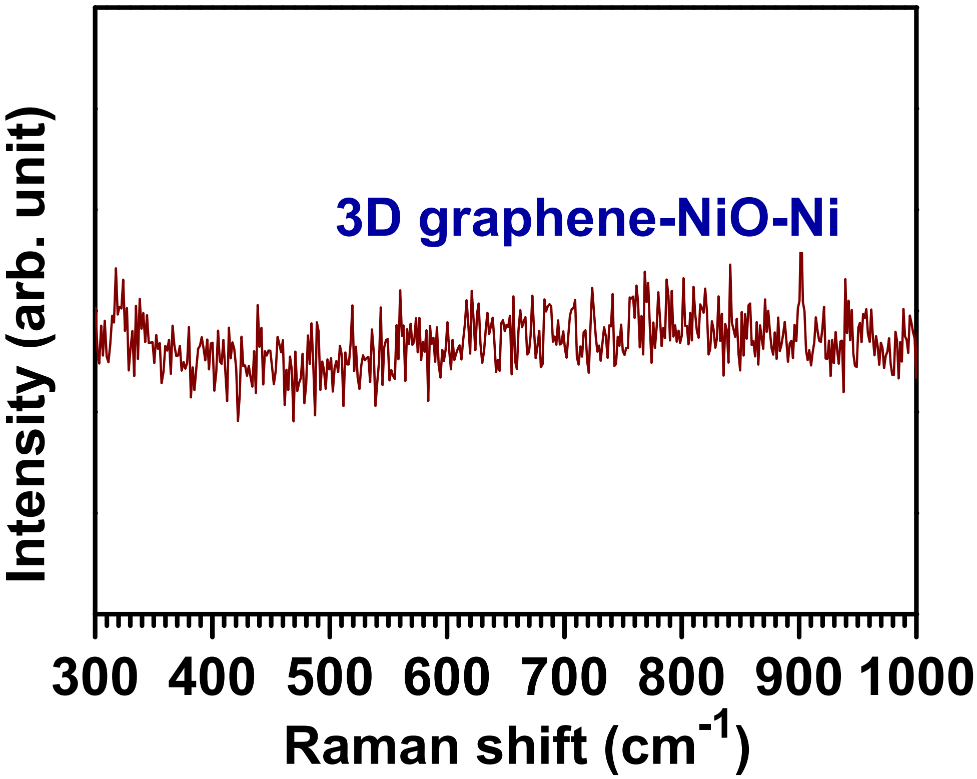

Figure S2. Raman spectra of the 3D graphene-NiO-Ni hybrid anode structure with wavenumber in the range of 300 to 1,000 cm^{-1}.

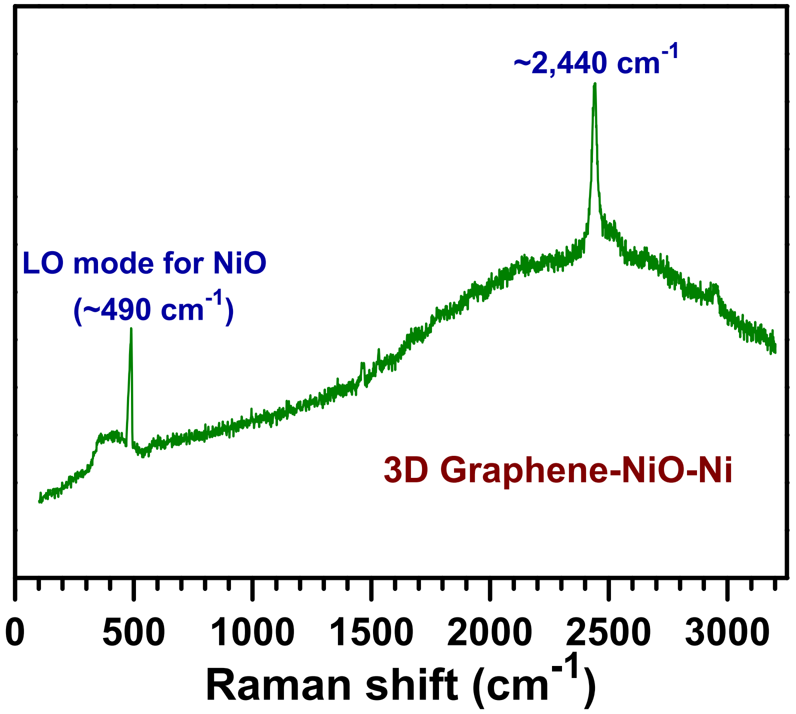

Figure S3. Raman data of the 3D graphene-NiO-Ni anode structure with wavenumber in the range of 100 to 3,200 cm^{-1}. Note that the 2D band peak for graphene is shifted from 2,700 to ~2,440 cm^{-1}, which is mainly due to the C-O bonding derived from 3D NiO-Ni substrate effect.

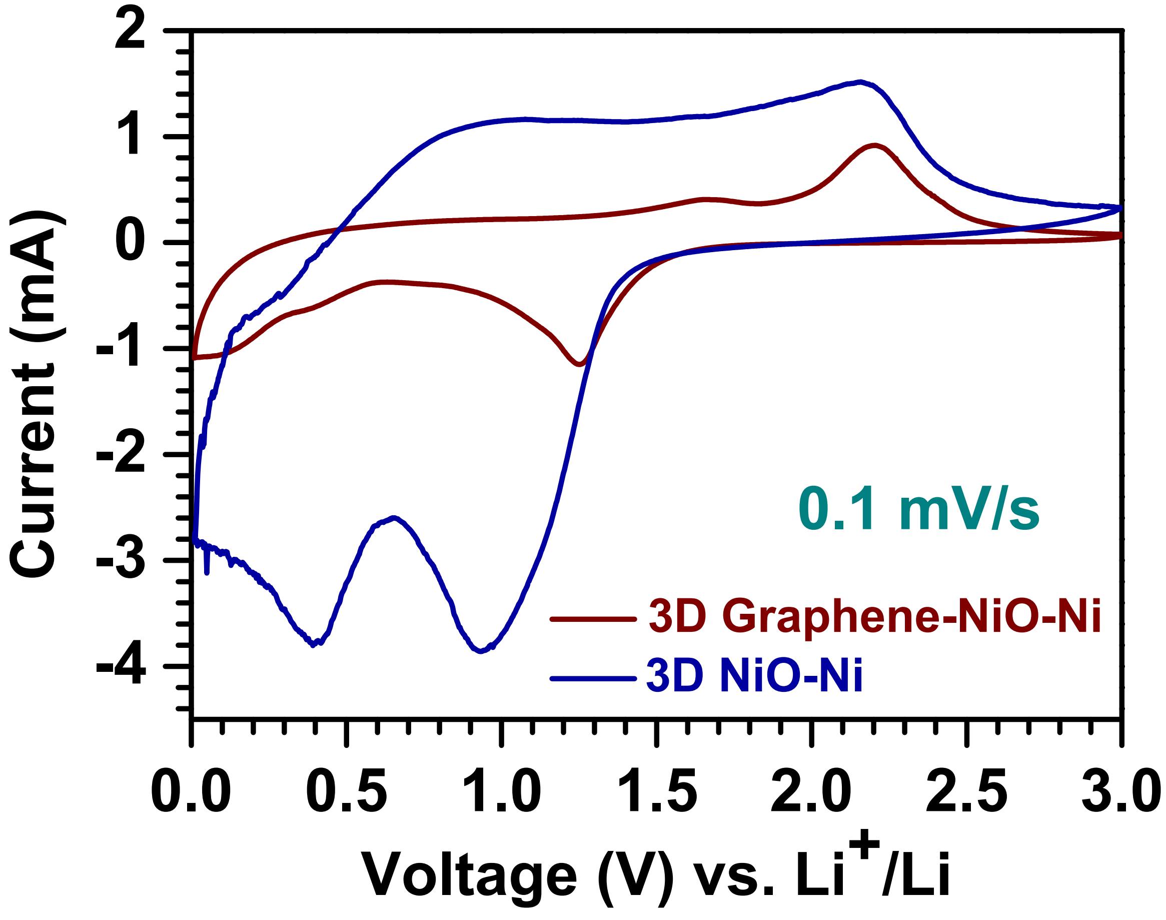

Figure S4. CVs of the 3D graphene-NiO-Ni and 3D NiO-Ni anode samples in the voltage range of 0.01 to 3V at a scan rate of 0.1 mV s^{-1} for the third cycle.
